# Overall survival and time trends in breast and cervical cancer incidence and mortality in the Regional Health District (RHD) of Barretos, São Paulo, Brazil

**DOI:** 10.1186/s12885-018-4956-7

**Published:** 2018-11-07

**Authors:** Allini Mafra da Costa, Dana Hashim, José Humberto Tavares Guerreiro Fregnani, Elisabete Weiderpass

**Affiliations:** 1 Hospital-Based Cancer Registry, Barretos Cancer Hospital, Rua Antenor Duarte Vilela, 1331, Dr. Paulo Prata, Barretos, São Paulo 14784-400 Brazil; 2 Population-Based Cancer Registry of Barretos Cancer Hospital, Barretos, São Paulo 14784-400 Brazil; 3 Institute of Education and Research, Barretos Cancer Hospital, Barretos, São Paulo 14784-400 Brazil; 4 Department of Research, Cancer Registry of Norway, Institute of Population-Based Cancer Research, Oslo, Norway; 5 Department of Medical Epidemiology and Biostatistics, Karolinska Institutet, Stockholm, Sweden; 6 Department of Community Medicine, Faculty of Health Sciences, University of Tromsø, The Arctic University of Norway, Tromsø, Norway; 7 Genetic Epidemiology Group, Folkhälsan Research Center, Helsinki, Finland

**Keywords:** Breast neoplasms, Gynaecologic neoplasms, Breast cancer, Cervix cancer, Epidemiology of chronic diseases, Mortality, Incidence, Trends

## Abstract

**Background:**

Breast and cervical cancers represent a significant cause of morbidity and mortality among women. The purpose of this study was to analyse the survival and time trends in two of the most common female cancers in the Regional Health District (RHD) of Barretos, São Paulo, Brazil.

**Methods:**

From 2000 through 2015, we calculated the breast and cervical cancer incidence and mortality rates per 100,000 women who were age-standardized to the world population. We obtained the time trends using the Joinpoint Regression software. We estimated the overall survival rates using the Kaplan-Meier methods.

**Results:**

The age-standardized rates (ASR) for incidence of breast cancer increased annually, with an average annual percentage change (AAPC) of 4.3 (95% Confidence Interval (CI): 2.4 to 6.3) for invasive breast cancer and 10.2 (95% CI: 6.1 to 14.5) for in situ breast cancer. The mortality rates for invasive breast cancer decreased with an AAPC of 0.2 (95% CI: -1.9 to 2.4). The ASR incidence of invasive cervical cancer showed an AAPC of − 1.9 (95% CI: -4.7 to 0.9). For in situ cases, the ASR showed an AAPC of 9.3 (95% CI: 3.3 to 15.7). The ASR mortality for cervical cancer showed an AAPC of − 5.3 (95% CI: -9.5 to − 0.8). The Kaplan-Meier analysis indicated 5-year overall survival rates of 74.3% for breast cancer and 70.7% for cervical cancer.

**Conclusions:**

The incidence of in situ and invasive breast cancer is increasing, while the mortality rates remain stable. We observed an increase in the incidence of in situ cervical cancer and a decrease in invasive incidence rates during the study period, and we noted that the cervical cancer mortality significantly declined during the study period.

**Electronic supplementary material:**

The online version of this article (10.1186/s12885-018-4956-7) contains supplementary material, which is available to authorized users.

## Background

Breast and cervical cancers are a significant cause of morbidity and mortality among women. Breast cancer is the most frequently diagnosed cancer (1.7 million) and the leading cause of cancer death (521,900 deaths) among women worldwide. Cervical cancer ranks fourth as the most frequently diagnosed type of cancer (527,600 new cases) among women worldwide, while in low- to middle-income countries, it is the second most commonly diagnosed type of cancer and the third leading cause of cancer death (265,700 deaths) among women [1].

There is a substantial variation in the incidence rates of breast and cervical cancers in different countries that may reflect the differences in the availability of early detection and screening services, as well as the differences in risk factors [1–3]. Mammography screening can often detect breast cancer at an earlier stage when treatment is more efficient, and the cure rate is higher, thereby decreasing mortality in a population [4]. For cervical cancer, such variation may be explained by the differences in the availability of screening techniques, allowing the detection and removal of precancerous lesions and the human papillomavirus (HPV) infection [1, 3, 5, 6]. In high-income countries, the Pap smear test is the most common screening method for cervical cancer. In low-resource countries, the most efficient and cost-effective screening techniques are visual inspection using acetic acid and HPV tests [7]. HPV vaccines Gardasil [Merck and Company, Whitehouse Station, NJ] and Cervarix [Glaxo Smith Kline, Brentford, UK] are available for protection against exposure to the most common risk of HPV for cervical cancers.

In Brazil, few studies have evaluated temporal cancer trends. Of these studies, Ayres et al. [8] showed an increasing trend of in situ and invasive cervical cancer in 4 capitals of Brazil in the period between 1990 and 2004. Lima et al. [9] showed an increasing incidence trend for breast cancer in the mid-sized northeastern Brazilian city, Aracaju, from 1996 to 2006. Several studies evaluating the temporal mortality trend showed a cervical cancer decrease and an increase or stable rate of the breast cancer over time [10–12].

The incidence of cancer can be evaluated using Population-Based Cancer Registries. There are 26 official cancer registries in operation (at least for one consolidated year) in Brazil, of which 19 are located in capitals, one in the Federal District, one with state coverage and another five in non-capital cities [13]. The Ministry of Health through the Brazilian National Cancer Institute (Instituto Nacional do Câncer – INCA) has been advancing the population-based cancer registry since its creation in the 1960s, focusing on non-capital cities, to establish how this chronic disease behaves in the interior of the country. To date, there have been no previous evaluations of the incidence and mortality trends in the Barretos region. We analysed the overall survival and time trends in the incidence of breast and cervical cancers and the mortality rate (2000–2015) among women using population-based cancer registry data for the RHD of Barretos, São Paulo state, Brazil.

## Methods

The current study was to describe the cancer incidence and mortality time trends. The State of São Paulo (Brazil) is administratively divided into 17 Regional Health Districts (RHDs). The RHD is responsible for coordinating activities at the regional level and promoting inter-sectorial articulation, with municipalities and civil society organisations [14]. RHD-V (Additional file 1: Figure S1) comprises 18 cities located in the Northeast of State of São Paulo State (Altair, Barretos, Bebedouro, Cajobi, Colina, Colômbia, Guaíra, Guaraci, Jaborandi, Monte Azul Paulista, Olímpia, Severínia, Taiaçu, Taiúva, Taquaral, Terra Roxa, Viradouro, and Vista Alegre do Alto), including both rural and urban populations, with 434,939 inhabitants (2017). According to the medium human development index (HDI), which comprises gross national income, education attainment, and life expectancy at birth, Brazil is high in the ranks [15]. The Barretos Cancer Hospital is responsible for the activities of the Population-Based Cancer Registry covering RHD-V, suitably called the “Population-Based Cancer Registry of Barretos Cancer Hospital.”

The Population-Based Cancer Registry of Barretos Cancer Hospital actively collected cancer cases from public and private sources such as hospitals, diagnostic and treatment clinics, pathology laboratories, units that provided comprehensive cancer treatment and followed the cancer classification rules recommended by the International Agency for Research on Cancer (IARC) as defined by the INCA. The methods of diagnosis were as follows: histology, cytology, imaging, clinical and laboratory evidence, and surgical findings. There was a careful review to verify the case data source as RHD of Barretos. Case duplicity was managed by Cancer Registry software [16] (SisBasePop, developed by INCA), which checked the information in several sources and databases. Classification and coding were performed according to the International Classification of Diseases for Oncology, 3rd edition (ICD-3) [17]. The International Classification of Diseases, 10th edition (ICD-10) was used as a coding standard for incidence and mortality. The topography considered was C50 (invasive breast cancer), D05 (in situ breast cancer), C53 (invasive cervical cancer) and D06 (in situ cervical cancer).

We calculated breast and cervical cancer incidence rates from 2000 to 2015 using data from the Population-Based Cancer Registry of Barretos Cancer Hospital, and breast and cervical cancer mortality rates from 2000 to 2015 using data from the Official Federal Database (DATASUS) (http://datasus.saude.gov.br/). The population references we used were based on the age distribution for the 18 cities belonging to the RHD-V (2000–2010). The DATASUS website provided intercensal estimates.

All data were stored in “.csv” format, which allowed for versatile data management using Microsoft Excel 2013 (Microsoft Corporation 2013) and Joinpoint Regression software, version 4.5.0.1 (June 2017; Statistical Methodology and Applications Branch, Surveillance Research Program, National Cancer Institute, EUA). Both incidence and mortality rates per 100,000 women were age-standardized, using the direct method, according to the world population [18–20]. A mortality-to-incidence ratio of ASRs was obtained to assess the burden of these two cancers. The time trends were obtained by calculating the average annual percentage change (AAPC) and 95% confidence interval (CI) for breast and cervical cancer incidence and mortality using log-linear Poisson regression. The overall survival (OS) rates were estimated in months of life accrued using the Kaplan-Meier method, and survival was defined as the period from the date of diagnosis to the time of death or the date at which information was last obtained from the patient. For the analysis, the event of interest was death. Cases that were alive were censored. Log-rank test was used to compare the survival curves. Cox regression estimated the hazard ratios. The chi-squared test was used to analyse by period and clinical stage the proportional differences in cases and deaths. For statistical analysis, we used IBM® SPSS® Statistics 20.0.1 software for Windows (IBM Corporation, Route 100, Somers, NY). The level of statistical significance was set at 0.05 for all analyses.

## Results

During the study period, 2110 new incident cases of breast cancer and 978 cases of cervical cancer were reported to the registry. A total of 477 breast and 136 cervical neoplasms deaths were detected in the mortality database. The characteristics of incident cases are shown in Table 1.

**Table 1:** Characteristics of the incidence of breast and cervical cancer cases according to Population-Based Cancer Registry of Barretos Cancer Hospital, São Paulo, Brazil, years 2000–2015

Variable	Breast cancer		Cervical cancer	
	N	%	N	%
Age at diagnosis				
< 30 years	22	1.0	219	22.4
30–39 years	128	6.1	224	22.9
40–49 years	502	23.8	189	19.3
≥50 years	1458	69.1	346	35.4
School level^a^				
Illiterate	158	7.8	82	8.8
<High school	1292	63.9	676	72.4
High school	295	14.6	109	11.7
College	276	13.7	67	7.2
Year of diagnosis				
2000–2004	410	19.4	242	24.7
2005–2009	596	28.2	185	18.9
2010–2015	1104	52.3	551	56.6
Clinical stage of disease at diagnosis (breast cancer: TNM; cervical cancer: FIGO)^a^				
In situ	338	16.5	619	65.9
I	468	22.9	84	8.9
II	649	31.8	91	9.7
III	405	19.8	94	10.0
IV	183	9.0	51	5.4
Total	2110	100	978	100

^a^Numbers in these variables do not add up to the overall total number of cases due to the missing values

**Fig. 1:**
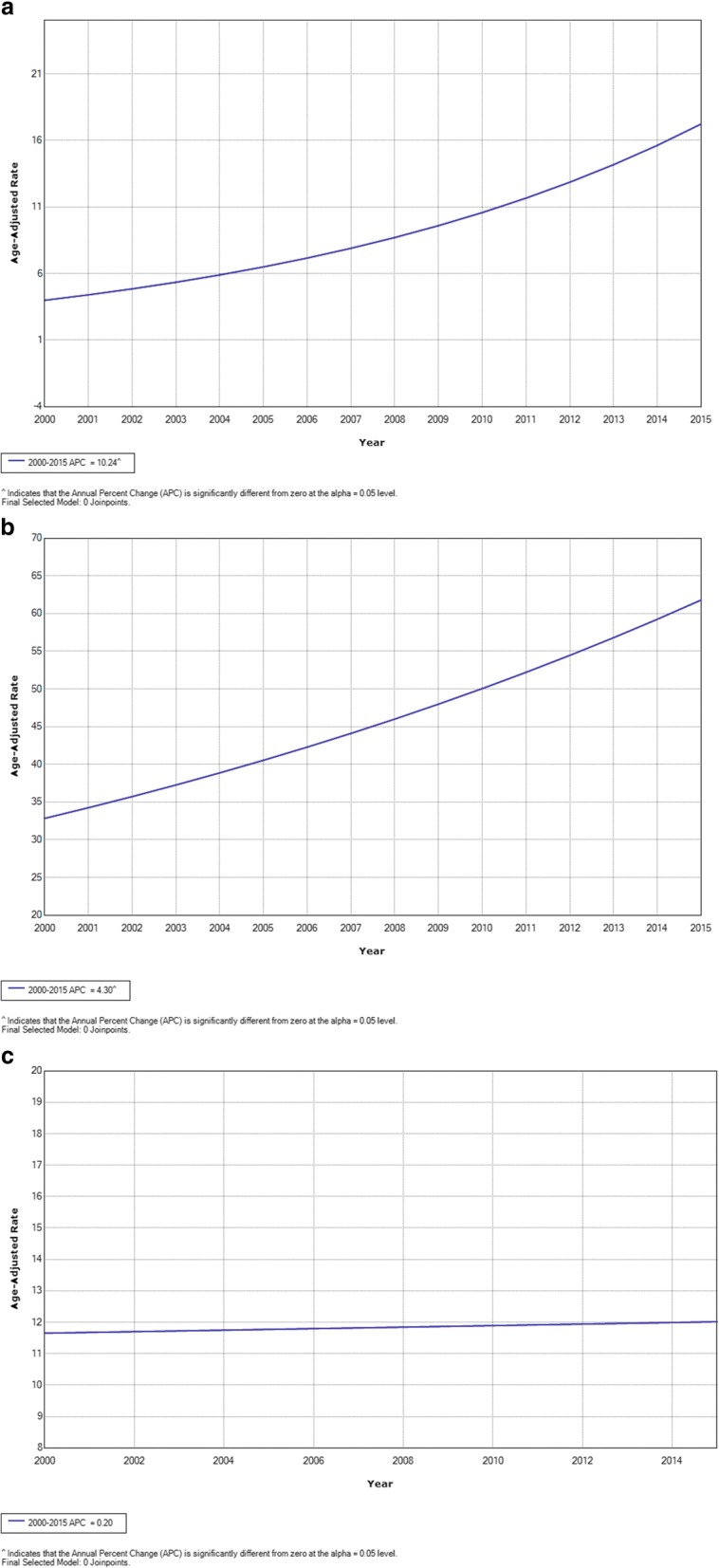
Joinpoint analysis of breast cancer, years 2000–2015. **a** In situ incidence; (**b**) invasive incidence; (**c**) mortality

**Fig. 2:**
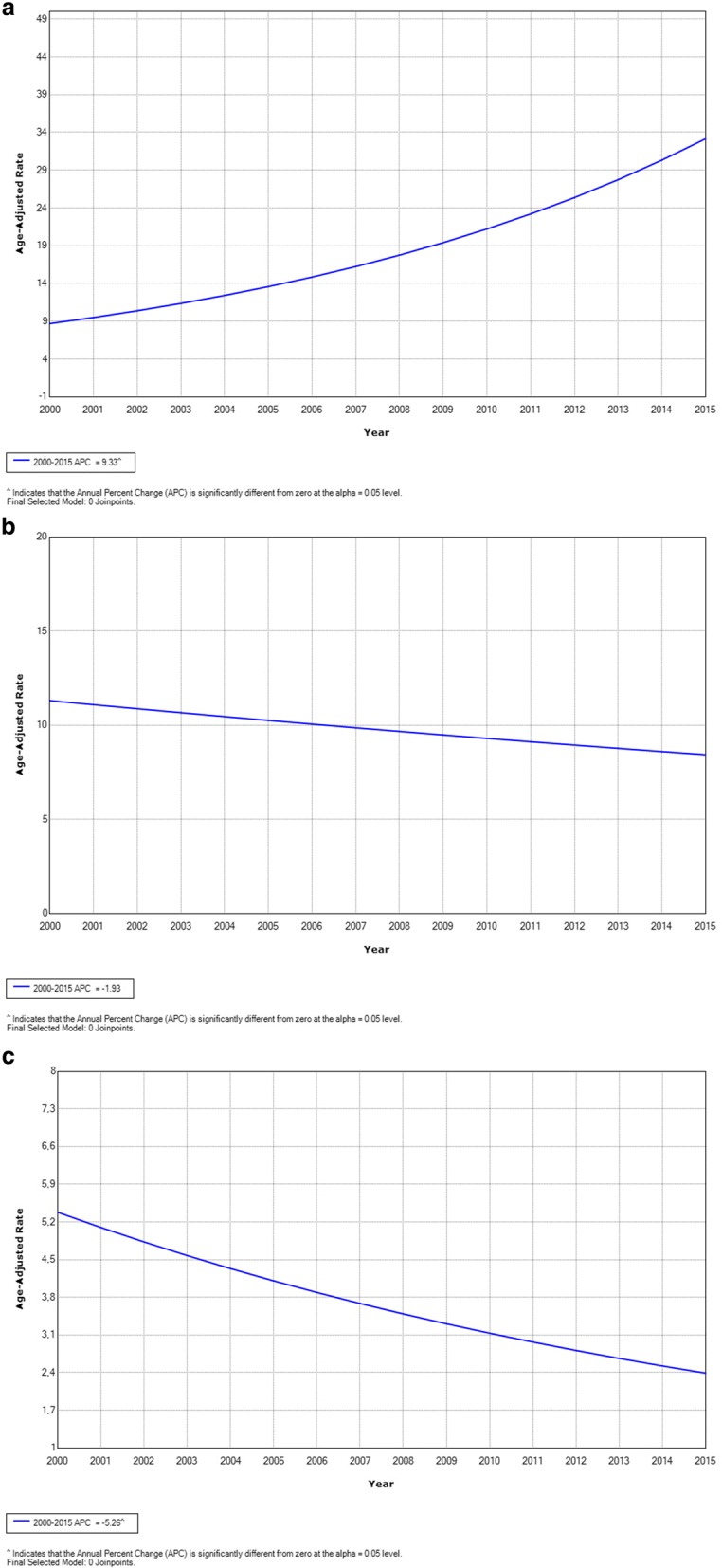
Joinpoint analysis of cervical cancer, years 2000–2015. **a** In situ incidence; (**b**) invasive incidence; (**c**) mortality

The incidence and mortality rates per 100,000 women are shown in Table 2, and the incidence and mortality trend curves are displayed in Figs. 1 and 2. The incidence rates for in situ breast cancer increased from 1.55/100,000 in year 2000 to 16.04/100,000 in year 2015 (AAPC: 10.2; 95% CI: 6.1 to 14.5) and for invasive breast cancer, the incidence rates increased from 29.09/100,000 in year 2000 to 64.09/100,000 in year 2015 (AAPC: 4.3; 95% CI: 2.4 to 6.3). Age-standardized incidence rates increased significantly. The mortality rates for invasive breast cancer were 10.4/100,000 in year 2000 and 14.00/100,000 in year 2015 (AAPC of 0.2; 95% CI: -1.9 to 2.4). The age-standardized incidence rates for in situ cervical cases were 10.90/100,000 in year 2000 and 24.84/100,000 in year 2015 (AAPC: 9.3; 95% CI: 3.3 to 15.7). For invasive cervical cancer, the age-standardized incidence rates were 10.67/100,000 in year 2000 and 8.87/100,000 in year 2015 (AAPC: -1.9; 95% CI: -4.7 to 0.9). For cervical cancer mortality, the age-standardized incidence rates varied from 4.32/100,000 in year 2000 to 3.13/100,000 in year 2015 (AAPC: -5.3; 95% CI: -9.5 to − 0.8). The mean mortality-to-incidence ratio for the time series was 0.27 for breast cancer and 0.37 for cervical cancer.

**Table 2:** Breast and cervical cancer incidence rates and mortality rates per 100,000 women according to the Population-Based Cancer Registry of Barretos Cancer Hospital, São Paulo, Brazil, years 2000–2015

Year	Breast cancer				Cervical cancer			
	In Situ Incidence (D05)	Invasive Incidence (C50)	Mortality	M:I^a^	In Situ Incidence (D06)	Invasive Incidence (C53)	Mortality	M:I^a^
2000	1.55	29.09	10.04	0.34	10.90	10.67	4.32	0.40
2001	4.16	36.54	11.59	0.32	21.13	13.29	3.72	0.28
2002	4.30	34.51	9.52	0.28	11.00	13.42	3.37	0.25
2003	1.90	37.51	14.05	0.37	7.29	10.33	6.27	0.61
2004	4.10	45.08	17.46	0.39	5.87	12.79	7.28	0.57
2005	7.30	44.92	12.63	0.28	10.84	9.29	2.43	0.26
2006	11.02	56.30	10.28	0.18	8.11	9.75	5.81	0.60
2007	11.90	35.89	10.07	0.28	7.51	7.23	4.01	0.55
2008	5.55	36.52	10.35	0.28	4.51	5.72	2.10	0.37
2009	8.34	34.20	9.43	0.27	8.67	6.57	2.04	0.31
2010	10.21	48.02	11.27	0.23	11.74	5.98	2.00	0.33
2011	10.99	45.92	11.59	0.25	21.38	7.19	2.21	0.31
2012	14.78	61.61	10.01	0.16	35.00	11.82	1.86	0.16
2013	14.62	58.63	13.75	0.23	41.82	10.37	3.36	0.32
2014	13.96	61.70	11.74	0.19	33.01	10.75	2.78	0.26
2015	16.04	64.09	14.00	0.22	24.84	8.87	3.13	0.35
Average	8.79	45.65	11.74	0.27	16.47	9.62	3.54	0.37
AAPC (95% CI) *p*-value	10.2 (6.1; 14.5) ≤0.05	4.3 (2.4; 6.3) ≤0.05	0.2 (−1.9; 2.4) > 0.05	NA	9.3 (3.3; 15.7) ≤0.05	-1.9 (−4.7; 0.9) > 0.05	-5.3 (−9.5; −0.8) ≤0.05	NA

*ASR* age-standardized rate (world population), *M:I* mortality-to-invasive incidence ratio, *AAPC* average annual percentage change, *CI* confidence interval, *NA* not applicable

^a^Includes ratio of mortality-to-overall incidence of invasive breast or cervical cancer, respectively

The clinical stage of disease at the time of diagnosis (TNM) versus the year of diagnosis is shown in Additional file 1: Table S1. A considerable reduction in stage IV cervical cancer disease was observed from 23.5 to 9.2% by 2015. The Kaplan-Meier analysis indicated a 5-year OS rate of 74.3% for breast cancer and 70.7% for cervical cancer. The patients diagnosed in the period ranging from 2000 to 2004 had the lowest OS rate (breast cancer: *p* = 0.058; cervical cancer: *p* = 0.004); and clinical stages III/IV had the lowest OS rates (*p* < 0.001 for breast and cervical cancers). The distributions of cases according to the patients’ characteristics and OS are shown with more detail in Table 3, and the OS curves are shown in Additional file 1: Figures S2 and S3.

**Table 3:** Overall survival rates according to the Population-Based Cancer Registry of Barretos Cancer Hospital, São Paulo, Brazil, years 2000–2015

Variable	Breast cancer					Cervical cancer				
	Cases	Deaths	5-y OS	p^a^	HR (95% CI)	Cases	Deaths	5-y OS	p^a^	HR (95% CI)
Overall survival	2110	597	74.3	**–**	**–**	978	200	70.7	**–**	**–**
Year of Diagnoses										
2000–2004	410	204	67.1	0.058	Reference	249	90	68.3	0.004	Reference
2005–2009	596	194	77.5		0.797 (0.648; 0.980)	185	51	64.2		1.214 (0.848; 1.739)
2010–2015	1104	199	75.0		0.801 (0.645; 0.996)	551	59	73.4		0.652 (0.456; 0.932)
Clinical stage of disease at Diagnosis (Breast cancer: TNM; Cervical Cancer: FIGO)										
In situ	338	26	96.0	< 0.001	0.035 (0.023; 0.053)	619	26	93.6	< 0.001	0.023 (0.014; 0.038)
I	468	61	89.4		0.061 (0.045; 0.082)	84	14	87.3		0.051 (0.027; 0.095)
II	649	154	81.9		0.097 (0.077; 0.121)	91	29	61.0		0.133 (0.082; 0.216)
III	405	184	57.3		0.269 (0.216; 0.334)	94	63	34.3		0.362 (0.245; 0.536)
IV	183	154	12.3		Reference	51	46	5.1		Reference

^a^Kaplan-Meier; *HR* hazard ratio, *CI* confidence interval. 5-y OS, 5-year overall survival

## Discussion

We found an increase in the incidence of both in situ and invasive breast cancer as well as a decrease in invasive cervical cancer from 2000 to 2015. While in situ cervical cancer increased during the period, a reduction of cervical cancer mortality was observed. Improvements in survival and cancer burden as reflected in mortality-to-incidence ratios were observed for both cancers. These findings are exciting because both breast and cervical cancer screening programmes were implemented around the same period in the RHD of Barretos, compared to many high-income countries performing nationwide screening before the study period. Thus, the increased incidence for these two cancers and the decreased mortality for cervical cancer may have been primarily influenced by screening programmes although treatment access may have also played a role. There was an increase in early diagnosis (in situ cases) for breast and cervical cancers and a decrease in the mortality rate for cervical cancer.

In Brazil, INCA [2] estimated in 2016 that 56 cases of breast cancer and 16 cases of cervical cancer would occur in every 100,000 women. For the state of São Paulo, 69 cases of breast cancer and 9 cases of cervical cancer were estimated to occur in every 100,000 women. Taking an average of the last 3 years detailed in the present study, the RHD of Barretos showed an incidence rate similar to that predicted by INCA, 61 cases of breast cancer and 10 cases of cervical cancer in every 100,000 women. Likewise, previous studies observed a decrease in cervical cancer mortality throughout the period and a decline in breast cancer mortality from 1990 to 2010.

Breast cancer screening practices in Brazil include biannual mammography for women aged 50 to 69 years with nationwide follow-up as outlined by the Brazilian National Cancer Institute (Instituto Nacional do Câncer - INCA) [21]. The present study shows a significantly increased incidence rate for in situ and invasive breast cancer, especially after the opportunistic mammography screening programme of Barretos Cancer Hospital implementation that started in 2003 in the RHD of Barretos (São Paulo) [22]; coverage rates have been increasing over the years. During this period, there were also several government actions, including directives to reduce the target age group, and there was considerable controversy involving the Brazilian Society of Radiology and the Brazilian Society of Mastology in this regard. From 2010, this opportunistic mammography screening programme in the RHD of Barretos had a differential structure that approximated an organised screening programme; therefore, it was unique in Brazil. As a tertiary level hospital, the Barretos Cancer Hospital is responsible for conducting mammography screening, complementary tests, medical care, diagnosis, treatment, and follow-up of patients. Mammograms can be performed either at the Department of Prevention (Barretos Cancer Hospital) or at the Mobile Health Unit that regularly visits the cities of the region sequentially, remaining for 3–5 days in each town. In this opportunistic mammography screening programme in the RHD of Barretos, women ranging in age between 40 and 49 years are offered annual mammography, while women ranging in age between 50 and 69 years are provided biannual mammography. While it is likely that higher screening coverage played a role in the increase in breast cancer incidence, the reductions of invasive breast cancer mortality-to-incidence ratio and survival rate during the study period also likely reflect better cancer outcomes for these patients and higher treatment availability in the Barretos RHD.

While some of the increase in breast cancer incidence rates can be explained by increased surveillance, the increase in invasive cancer (4.3%) cannot be explained by screening alone. Worldwide breast cancer incidence has increased by over 20% since GLOBOCAN 2008 [1, 23], and many of the countries with increased breast cancer rates, such as the UK and US, already had breast cancer screening programmes. This trend of increasing breast cancer incidence in the range of 4% to 10% has also been observed in other countries that have transitioned from low income to middle income (such as Philippines, Columbia, and Costa Rica) or from middle income to high income (such as Israel and the Czech Republic) [20, 24]. These findings might be attributable to lifestyle changes associated with higher economic development, such as women having fewer children and prolonged age at first birth, and increased use of oral contraception [25]. Other lifestyle changes can also be due to increases in the prevalence of lifestyle risk factors, such as obesity and low levels of physical activity [26].

Our present study indicates a significant increase in the incidence of cervical cancer and a decrease in mortality due to cervical cancer in the RHD of Barretos in recent years. Improvements in Brazilian government strategies most likely have influenced these trends as well as the installation of the Organized Cervical Cancer Screening Program in the RHD of Barretos. Cytology using Pap smear tests is the screening method offered to all women aged 25–64 years who are sexually active according to the Brazilian Guidelines for Cervical Cancer Screening [21]. Since 1994, the Barretos Cancer Hospital has introduced the use of mobile units that offer cervical cancer screening to the target population of the RHD [27]. From 2012, women aged between 25 and 64 years are invited annually to participate in the cervical cancer prevention programme via municipality-specific health service units [28]. Letters are mailed to all women who were already registered in a database and who were not up-to-date on their Pap smear, as well as women for whom a Pap smear should be repeated when the mobile unit is present in their municipality [28]. The invitations are sent to women following the screening protocol, and the exams are routinely conducted at intervals of 3 years after two annual negative exams [28]. A systematic follow-up of the women participating in the cervical cancer screening programme is performed using software developed by the Barretos Cancer Hospital for this purpose [28]. However, this government lacks reliable information on coverage rate, which is essential for programme monitoring, and future studies are needed to monitor the prevalence of abnormal cytology screening results and to quantify the effectiveness of cervical cancer screening in Brazil since the introduction of this screening programme.

A significant strength of this study is the multiple measures taken to accurately capture the cancer incidence and mortality of over 200,000 women living in the Barretos RHD. As the Population-Based Cancer Registry data of Barretos Cancer Hospital were retrieved from several different sources of information, some cases could be found in more than one information source. Extra care was taken to avoid duplication of registered cases. Many patients declared their residence in Barretos, which is the headquarters of one of the largest centres for cancer treatment in Brazil, which could artificially increase cancer incidence rates in the region. We identified and confirmed cases from the geographical area of the cancer registry by reviewing the primary sources of information to mitigate this issue. The mortality rates were calculated from death records of the DATASUS. The cause of death registration in this database has been described as being imprecise, mainly in rural areas, which can jeopardise our conclusions. The indicators would be higher than those officially disclosed if corrections were made regarding undefined deaths and cases classified as a non-specified area of the uterus. However, official data were used, and all possible effort was aimed at improving the quality of information.

## Conclusion

Our study demonstrates that breast and cervical cancer incidence rates were increasing over time between 2000 and 2015 in the RHD of Barretos (São Paulo). The mortality trends did not significantly increase for breast cancer, while for cervical cancer there were statistically significant decreases in mortality over time, likely due to the implementation of screening and the availability of treatment. For breast cancer, the increasing incidence trend could not be explained by screening alone, but it may be due to changes in the prevalence of lifestyle risk factors in the Barretos RHD. The survival rate of these women improved over time.

## Additional file

Additional file 1: **Table S1.** Comparison between clinical stage of disease (TNM) a year of diagnoses according to Population-Based Cancer Registry of Barretos Cancer Hospital, São Paulo, Brazil, years 2000–2015. **Figure S1.** Area of coverage of the RHD of Barretos (RHD-V). **Figure S2.** Breast cancer overall survival. (a). Overall survival; (b). Overall survival by year of diagnoses; (c). Overall survival by clinical stage. **Figure S3.** Cervical cancer overall survival. (a). Overall survival; (b). Overall survival by year of diagnoses; (c). Overall survival by clinical stage. (DOCX 451 kb)

## Abbreviations

AAPC: Average annual percentage change
ASR: Age-standardized rate
ASR: Age-standardized rates
BCH: Barretos Cancer Hospital
CI: Confidence Interval
DATASUS: Department of Informatics of the Unified Health System - Official State Database (Brazil)
HDI: Human Development Index
HPV: Human papillomavirus
HR: Hazard ratio
IBGE: Instituto Brasileiro de Geografia e Estatistica (Brazilian Institute of Geography and Statistics)
INCA: Instituto Nacional do Câncer (Brazilian National Cancer Institute)
M:I: Mortality-to-invasive incidence ratio
N: Number of cases
NA: Not applicable
OS: Overall survival
RHD: Regional Health District
