# Supplementary material for: Overall survival and time trends in breast and cervical cancer incidence and mortality in the Regional Health District (RHD) of Barretos, São Paulo, Brazil
Source: BMC Cancer. 2018 Nov 7;18:1079. doi: 10.1186/s12885-018-4956-7 (PMC6223073; doi:10.1186/s12885-018-4956-7)
Supplement: Supplementary file 1 — Table S1. Comparison between clinical stage of disease (TNM) a year of diagnoses according to Population-Based Cancer Registry of Barretos Cancer Hospital, São Paulo, Brazil, years 2000–2015. Figure S1. Area of coverage of the RHD of Barretos (RHD-V). Figure S2. Breast cancer overall survival. (a). Overall survival; (b). Overall survival by year of diagnoses; (c). Overall survival by clinical stage. Figure S3. Cervical cancer overall survival. (a). Overall survival; (b). Overall survival by year of diagnoses; (c). Overall survival by clinical stage. (DOCX 451 kb) [file 12885_2018_4956_MOESM1_ESM.docx]

**Suplementary file**

**Suplementary Table 1.** Comparison between clinical stage of disease (TNM) a year of diagnoses according to Population-Based Cancer Registry of Barretos Cancer Hospital, São Paulo, Brazil, years 2000-2015.

| **Clincial stage of disease**  **at Diagnosis (TNM)*** | **Breast cancer** | | | |  | **Cervical cancer** | | | |
| --- | --- | --- | --- | --- | --- | --- | --- | --- | --- |
|  | **2000-2004** | **2005-2009** | **2010-2015** | **p** |  | **2000-2004** | **2005-2009** | **2010-2015** | **p** |
| In situ | 32 (8.1) | 103 (17.7) | 203 (19.1) | <0.001 |  | 118 (51.3) | 93 (52.8) | 408 (76.5) | <0.001 |
| I and II | 237 (59.7) | 333 (57.2) | 547 (51.4) |  |  | 58 (25.2) | 41 (23.3) | 76 (14.3) |  |
| III and IV | 128 (32.2) | 146 (25.1) | 314 (29.5) |  |  | 54 (23.5) | 42 (23.9) | 49 (9.2) |  |
| **Total** | **397 (100.0)** | **582 (100.0)** | **1,064 (100.0)** |  |  | **230 (100.0)** | **176 (100.0)** | **533 (100.0)** |  |

* Numbers in these variables do not add up the overall total number of cases due to the missing values.

**
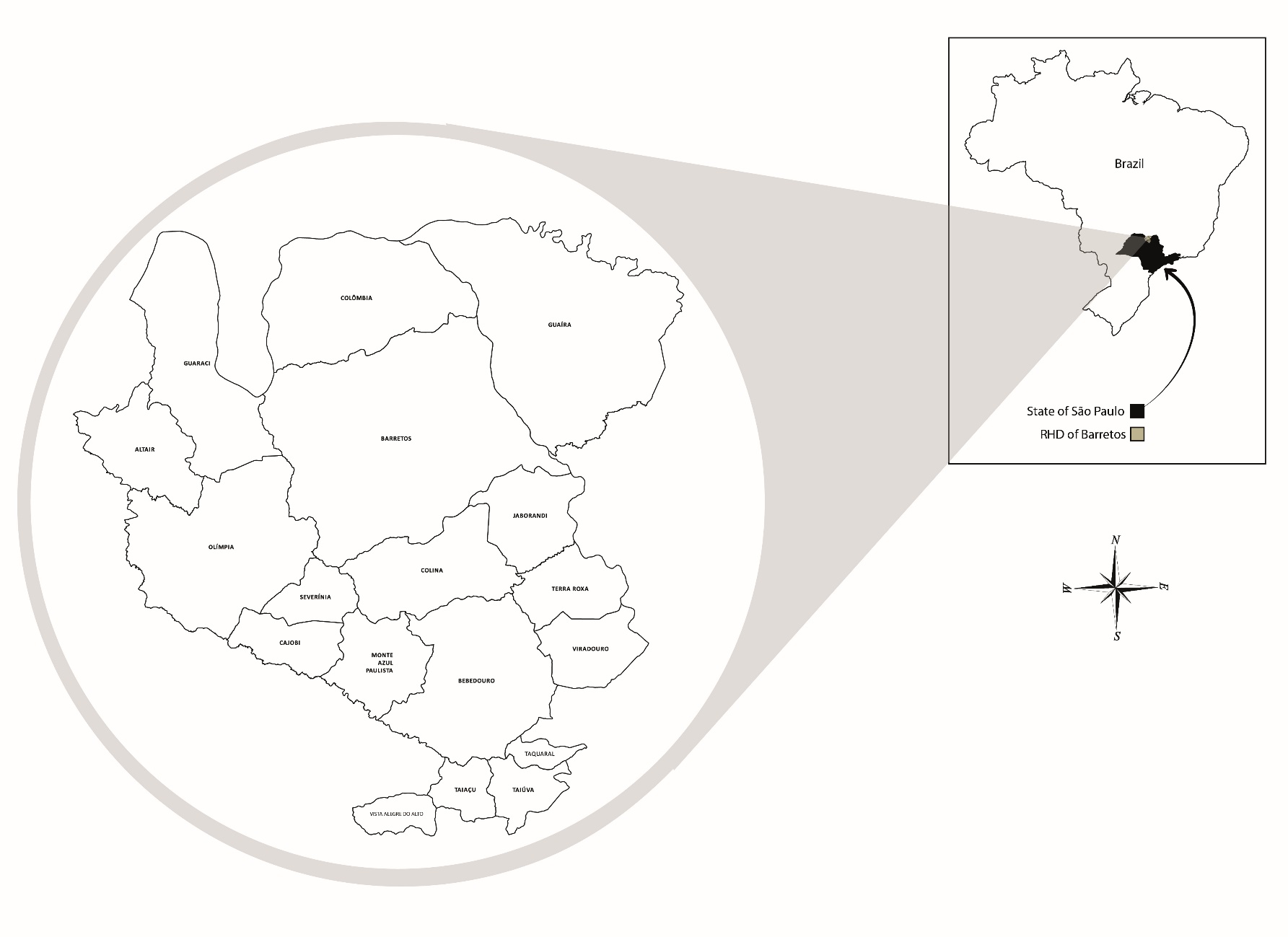
**

**Suplementary Figure 1.** Area of coverage of the RHD of Barretos (RHD-V).


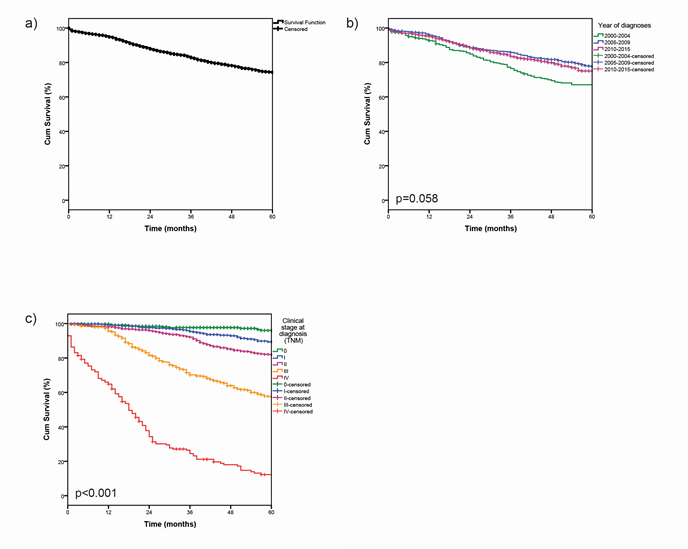


**Suplementary Figure 2.** Breast cancer overall survival. (a). Overall survival; (b). Overall survival by year of diagnoses; (c). Overall survival by clinical stage.


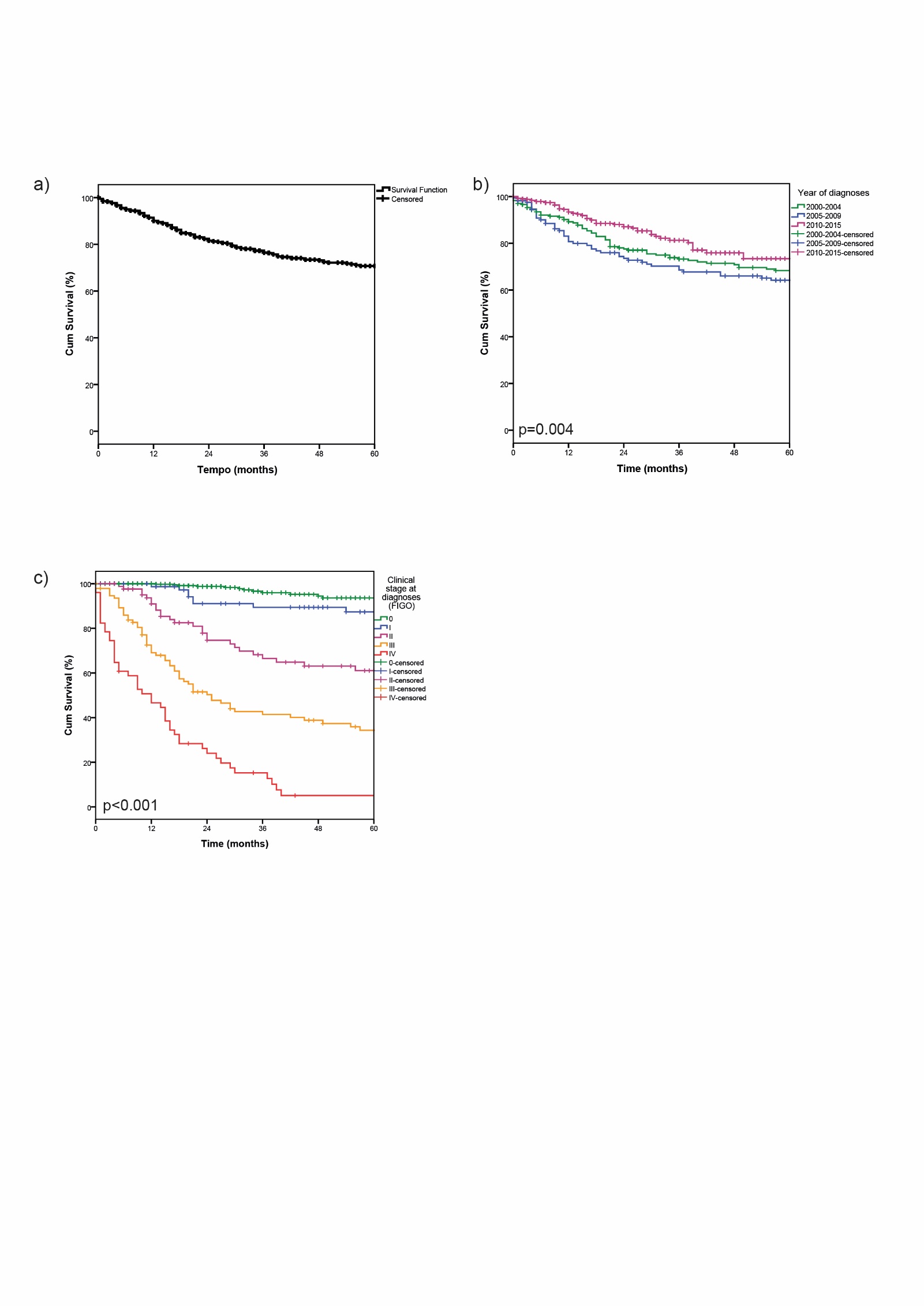


**Suplementary Figure 3.** Cervical cancer overall survival. (a). Overall survival; (b). Overall survival by year of diagnoses; (c). Overall survival by clinical stage.
